# Optimizing gas entry–exit capacity utilization under uncertainty

**DOI:** 10.1007/s10287-025-00552-3

**Published:** 2026-01-19

**Authors:** Berend Markhorst, Ruurd Buijs, Ruud Egging-Bratseth, Rob van der Mei

**Affiliations:** 1 Centrum Wiskunde en Informatica, Amsterdam, The Netherlands; 2 Vrije Universiteit, Amsterdam, The Netherlands; 3 SINTEF Industry, Trondheim, Norway

**Keywords:** Stochastic programming, Entry–exit capacity markets, Norwegian natural gas, Capacity allocation under uncertainty

## Abstract

Natural gas is vital to Europe’s energy system, with Norway supplying 30% of European gas demand. Effective management of entry–exit capacity in the Norwegian network can enhance market efficiency and energy security, but is far from trivial due to uncertain demand and prices. This study develops a stochastic programming model to determine optimal capacity allocation under uncertainty, with a focus on scalability. Concerned about network stability, operators tend to be risk averse in deviating from their initial decisions when allocating bookable capacities. We use our model in a case study on Norway’s gas pipeline network and find that moderating risk aversion can yield considerable system welfare gains. Additionally, we give insights into the system bottlenecks for policymakers and industry stakeholders and show the value of flexibility in this context. Finally, we provide a comprehensive dataset to advance future research.

## Introduction

Natural gas is one of the most prominent energy carriers in Europe and plays a vital role in Europe’s energy system today. It contributes approximately 22% to the EU’s primary energy consumption and serves around 40% of European households (Agency for the Cooperation of Energy Regulators 2025). Europe relies heavily on imports to meet its gas demand; about 80% of gas demand is covered by imports (Agency for the Cooperation of Energy Regulators 2025). This import dependency has created vulnerabilities, which have become more prominent in recent years due to geopolitical tensions (European Central Bank 2023). Besides, natural gas is important for the future development of the electricity (Ordoudis 2018) and hydrogen system (Durakovic 2024).

Norway is Europe’s largest natural gas supplier meeting about 30% of Europe’s gas demand (Reuters 2025). As the EU seeks to diversify its energy sources away from Russian supply, Norway’s stable and reliable gas exports are increasingly valuable. The total production of natural gas in 2024 in Norway amounted to 120 billion standard cubic meter (bcm), and, yielded an export value of over €40 billion in 2023 (Norwegian Petroleum 2025a). According to Norwegian Petroleum (2025c), 94 oil and gas fields were in production at the end of 2024, of which Troll, Johan Sverdrup, and Snøhvit are the essential fields. The Norwegian gas network connects key European markets with a handful of key pipelines including: Europipe I and II, and Norpipe (to Germany), Langeled (to the UK), Zeepipe (to Belgium), and Franpipe (to France) (Gassco 2024). At present, 25 companies are involved in the production of gas and oil on the Norwegian shelf (Norwegian Petroleum 2025b).

There are several reasons why natural gas flows will become less stable in future years. Annual European natural gas demand has been declining and is expected to continue to do so (International Energy Agency 2025). Additionally, intermittency of renewable energy sources (RES) generation can spill over to the natural gas system when gas fired power generation demand is used in low RES generation high electricity load periods. Suppliers will want to respond to short-term demand and price developments. To facilitate the uptake of hydrogen in the European system as part of the broader energy transition, hydrogen may be blended into natural gas and significant parts of the existing gas infrastructure may be repurposed for dedicated hydrogen transport. Norway can play a significant role in this transition in several ways with optimal capacity management to increase available capacity within the transmission system. Optimizing the use of this infrastructure could improve market efficiency for both natural gas and hydrogen and further bolster Norway’s position as a key energy partner to Europe.

Gas network capacity management in Europe has gone through several phases in recent decades, following various energy packages aimed at improving market efficiency. To give a broader perspective, in the following we briefly review the history of gas market liberalization in Europe, a transformative process that redefined the structure of the gas sector. This shift aimed to promote competition, enhance market transparency, and empower consumers by breaking down monopolistic barriers and fostering cross-border energy trade (Ciucci 2024).

The liberalization process began in the 1990 s, when the European Union (EU) introduced a series of energy packages designed to open national energy markets. The First Energy Package (EC 1998) laid the groundwork by requiring member states to provide third-party access to energy networks and establish independent regulatory authorities. However, competition remained limited, as incumbent energy companies retained significant control over infrastructure and supply.

The Second Energy Package (EC 2003) took a step further by granting consumers the right to choose their energy suppliers. It also marked a significant shift away from vertically integrated state-owned utilities and set the stage for increased cross-border trade and competition. However, full market integration remained a challenge due to continued dominance by national energy giants.

To address these persistent issues, the Third Energy Package (EC 2009) mandated the unbundling of energy generation and supply from transmission systems. This structural separation aimed to prevent conflicts of interest, ensuring that transmission network operators acted independently of supply companies. The package also strengthened the role of the Agency for the Cooperation of Energy Regulators (ACER) and enhanced transparency in pricing and market operations.

A cornerstone of market liberalization was the implementation of Third-Party Access (TPA) (EC 2024), which granted new entrants the right to use existing energy networks under transparent and non-discriminatory terms. This was essential to breaking down barriers to market entry and stimulating competition. Moreover, the introduction of the entry–exit system redefined how gas was traded and transported. By decoupling physical gas flows from commercial transactions, this system allowed market participants to book capacity at entry and exit points rather than along fixed routes, enhancing market flexibility and efficiency. Together, these reforms laid the foundation for a competitive and integrated European energy market (Ciucci 2024). They not only improved market transparency and efficiency but also ensured fairer consumer rights.

As buyers typically adjust periodic volumes to better match fluctuations in demand, their contracts typically include nomination flexibility (Ason 2022). Suppliers need entry–exit capacity to deliver gas to these buyers, which can be acquired from the TSO (the *primary* market) or the other suppliers (the *secondary* market). By giving suppliers the flexibility to trade in a secondary market, the gas network can be used more efficiently as it ensures a better alignment between supply and consumption. We will apply the notion of “flexibility” in different settings: besides (1) the flexibility of a supplier to trade in a secondary market, (2) the flexibility to store gas temporarily, and (3) the flexibility to adapt decisions over time to prices and demand changes.

As the TSO must manage gas flows at the pipeline level to ensure flow feasibility, when optimizing system capacity there are trade-offs between maximizing capacity availability, providing long-term certainty, and providing short-term flexibility for suppliers to book the entry–exit capacity. At the same time, the TSO must be able to manage unplanned disruptions in the network. To achieve this, TSOs apply safety margins when determining available capacity, accounting for both operational uncertainties and potential disruptions. All this, combined with natural gas demand and price uncertainty, leads to a complex capacity booking and allocation process, which is exacerbated by the decoupling of capacity bookings in the entry–exit system, and the need for feasible flows at the pipeline level. In this study, we address the following three research questions:

1. How does risk aversion affect capacity allocation and capacity availability in various strategic and operation stages?
2. What are the bottlenecks of the system and how can we solve them?
3. What is the value of booking-flexibility for storage facilities?

We focus on these questions when describing and analyzing the entry–exit capacity market under uncertainty using a stochastic programming model. To address uncertainty realistically, we propose a linear program which is a tractable and scalable method. Using this model, we conduct a case study on the Norwegian natural gas pipeline system perspective.

### Contributions

The contributions of this work are:

- We develop a stochastic programming model for the primary and secondary entry–exit capacity markets of natural gas with multiple suppliers, which endogenously determines how much entry and exit capacity the Transmission System Operator should make available so as to maximize the utilization rate of the network. Our model can optimize for sufficiently large scenario sets within a considerably small amount of time and yields better scalability compared to the other models proposed in the literature.
- We base our findings on a realistic case study that is more extensive and more detailed than used in previous work by Fodstad et al. (2015) and Grimm et al. (2019). The corresponding data set is available at Markhorst (2025).
- We identify bottlenecks in the Norwegian gas network and find that a less risk averse approach yields an increase of approximately 0.25% in the system profit.

The rest of this paper is structured as follows. Section 2 contains an outline of the current state of the literature. In Sect. 3, we will outline our problem and the market context giving rise to it. Thereafter, we introduce and detail our model in Sect. 4. Section 5 presents the results and discusses the implications of these findings within the context of Norway’s natural gas industry. Finally, Sect. 6 concludes the paper, summarizing the main contributions and insights, and proposing directions for future research.

## Literature

We provide an overview of literature pertaining to gas transport capacity booking and flow optimization as well as some illustrations for the computational complexity of such problems. This provides a backdrop for our research questions, and support for developing a scalable approach.

The literature in the domain of mathematical optimization for the natural gas networks is rich and diverse, covering topics such as the design and operation of its infrastructure (Hellemo 2016), pipeline capacity booking (Fodstad et al. 2015), pipe sharing (Zhao et al. 2024), or its transportation (Ríos-Mercado and Borraz-Sánchez 2015). Mathematical optimization models based on mixed-integer linear programming and nonlinear programming can help determine capacity, verify network abilities, and decide on network expansions, c.f., Fügenschuh et al. (2014), which is far from trivial. For example, the work of Schewe et al. (2020) explores the computational complexity of determining maximal technical capacities in the European gas market’s entry–exit system and finds that it is NP-hard in certain cases.

In Hellemo (2016), a model is developed to assess investments in infrastructure while accounting for uncertainties in the natural gas industry, such as fluctuating prices, demand, and resource quality. To address these issues, the authors develop optimization models that consider both short-term operational variability and long-term uncertainties. Applied to the Norwegian Continental Shelf, these models demonstrate substantial cost savings and improved decision-making for investments in natural gas production and transport capacities. In the work of Fodstad et al. (2015), the authors state that interruptible transportation services provide an innovative approach to enhance the flexibility and efficiency of natural gas networks. Unlike firm services, which guarantee capacity availability, interruptible services transport gas only when spare capacity exists. The authors show that integrating such services can (1) boost gas flow efficiency, (2) allow shippers to adapt to uncertainties flexibly, and (3) deliver economic benefits without compromising supply security. The authors of the current state-of-the-art in this field, Grimm et al. (2019) present a four-level model analyzing supplier-TSO interactions, inefficiency levels, and potential market design improvements. A “first-best benchmark model” is proposed to compare the idealized system against real-world scenarios, with simplifications suggested to enhance runtime while preserving key insights. Similarly, Böttger et al. (2022) addresses inefficiencies using a robust optimization approach to reduce a multilevel model to a single-level problem. The study finds that suboptimal network designs can lead to welfare losses and discrimination against smaller suppliers, emphasizing the importance of tailored pricing mechanisms and flexible policies. The technical complexities of gas flow dynamics further complicate market operations. In Hiller et al. (2018), the authors develop a stochastic optimization model for the European entry–exit market, integrating nonlinear and mixed-integer constraints. Their software system evaluates capacities and validates nominations using historical data, though computational speed poses a challenge, which differs from this work as we propose a scalable, linear method. Finally, we highlight two recent related works (Schewe et al. 2022; Grimm et al. 2023). The former tackles the multilevel structure of gas networks, including the physical dynamics of gas flow. The authors reformulate nonlinear flow problems into computationally feasible single-level models using convex constraints and integer variables. Applied to the Greek gas network, the approach effectively handles tree-shaped networks, introducing combinatorial constraints to accelerate calculations. Contrary to this work, which studies a relatively small gas network, we apply our method on a larger and realistic network, which represents the Norwegian Continental Shelf and contains connections to several European countries. Finally, the interplay of market power and pricing mechanisms is explored in Grimm et al. (2023). Here, a four-level model captures the strategic decisions of a monopolistic gas seller, TSO, and buyers. Reformulated into a single-level model, the study reveals that price discrimination can improve outcomes in congested networks, while uniform pricing remains effective in unconstrained scenarios. Despite the simplifications, computational challenges persist in larger networks with extended time horizons.

Table 1 shows an overview of the most relevant papers to this work including their properties. All works, except (Fodstad et al. 2015) focus on the entry–exit system and three works include a case study (Grimm et al. 2019; Schewe et al. 2022; Böttger et al. 2022), which use gas networks that are either heavily stylized or considerably small. In our work, we conduct a case study on a larger gas network. Due to the nature of the gas market, such as uncertainty in renewable energy (Durakovic et al. 2024), or gas prices and demand, stochasticity should be an inherent part for the analysis of this setting. However, the table contains only two published works that account for uncertainty. Then, only (Grimm et al. 2023) considers the case of a monopolistic supplier while all other works assume perfect competition. As explained by Grimm et al. (2023), multilevel approaches are usually required to model the sequential decision-making structure between the TSO and the suppliers, with the TSO setting explicit capacity boundaries to enforce that any submitted bid within these boundaries is feasible with regards to transportation. Also, due to the physics of gas flows (Weymouth 1912; Fügenschuh et al. 2015; Domschke et al. 2023) some constraints are nonlinear. More specifically, there are nonlinear properties in pressure dynamics in pipelines, compressor efficiency and gas quality management (Fodstad et al. 2015). Additionally, integer and binary variables are required to model specific decisions, such as the interruption of some firm booking in Fodstad et al. (2015) or the opening/closing of valves in Grimm et al. (2019). The last row of Table 1 shows the properties of this work and how it relates to the literature.

**Table 1:** Overview of key papers with relevant research properties

References	Topic	Case study?	Entry-exit?	Stochastic?	Perfect competition?	Gas physics?	Method
Fodstad et al. (2015)	Interruptible transportation services	$\checkmark$ (small network)	✗	$\checkmark$	$\checkmark$	✗(approximation)	Sequential method; contains a MILP
Grimm et al. (2019)	Unused network capacity and market design	✗	$\checkmark$	✗	$\checkmark$	$\checkmark$	Multilevel equilibrium model
Schewe et al. (2022)	Global optimization for the multilevel European gas market system	$\checkmark$ (small network)	$\checkmark$	$\checkmark$	$\checkmark$	$\checkmark$	MINLP
Böttger et al. (2022)	The cost of decoupling trade and transport	$\checkmark$ (small network)	$\checkmark$	✗	$\checkmark$	$\checkmark$	Single-level mixed-integer quadratic problem
Grimm et al. (2023)	A tractable model for entry–exit market with market power	✗	$\checkmark$	✗	✗	$\checkmark$	Multilevel model. Under sufficient conditions a tractable single-level model
This work	Capacity utilization under uncertainty	$\checkmark$ (large network)	$\checkmark$	$\checkmark$	$\checkmark$	✗	LP

Our proposed method considers uncertainty in the entry–exit market for natural gas from a high-level perspective and is used in a case study with a large natural gas network. Because we are interested in such high-level insights, we do not require a detailed accounting for the pressure dynamics, compressor efficiency, or additional details regarding gas flow physics. As a result, this resolves all the previously mentioned nonlinear and integrality requirements in the literature.

In the model of Grimm et al. (2019), a bilevel structure is used to model the directive power of the TSO to guide the capacity bidding process by explicitly setting maximal entry and exit capacities on the network nodes in order to ensure transportation feasibility for all possible bids. As mentioned by Grimm et al. (2019), this manner of guaranteeing feasibility can lead to network inefficiencies, as it tends to underusing capacity in several scenarios. This is due to the elimination of technically feasible bids that cannot be submitted because of the strict capacity limits, which disallow exploitation of the interplay between different nodes when allocating (maximum) capacities. Furthermore, in the introduction of the same work, it is mentioned that these inefficiencies will become more and more problematic in the future as desired network utilization is expected to increase. In order to maintain tractability while adding uncertainty to the model, we propose a single level structure, which can be achieved by relaxing the assumption of strict capacity limits set by the TSO. In the single level structure, all bids for which a fully feasible transportation schedule can be derived are considered to be submittable, even if they cannot emerge in a setting where fixed capacity limits are set beforehand. Finally, contrary to Fodstad et al. (2015), we publish our dataset, which has a realistic network size, online (Markhorst 2025) to accelerate future research.

## Problem description

Similarly to most works in the literature, see Table 1 from Sect. 2, we assume a single-commodity market for natural gas where multiple production field operators (*suppliers*) are in perfect competition with one another. We study the network capacity management problem primarily from the perspective of the Transmission System Operator (TSO), which aims to maximize social welfare. This will, however, also require to find realistic strategies for the individual suppliers involved. Therefore we assume that all suppliers act as profit-maximizing entities.

To reflect the typical sequencing of capacity allocation in natural gas networks, we assume that the capacity planning of the network is done by sequentially taking decisions in three stages:

1. Long-term (months or years ahead)
2. Day ahead
3. Intraday

The planning decisions within each stage appertain to a set of fixed time blocks, that form a partition of a single day. In the final stage, the TSO is required to facilitate operation in accordance with the capacity and planning decisions corresponding to each of the time blocks in all stages. The TSO is tasked with finding a feasible operational plan of gas flow on the network in the first stage, long before the operational period. The TSO needs to ensure technical stability when operating the network, as suppliers and consumers count on continuous supply, and disruptions can have dramatic and costly consequences. Therefore, the TSO typically prefers to be in control of the situation, and is hesitant to allow large modifications in their operational planning as the operational period approaches. Depending on the risk preferences of the TSO, there are different levels of strictness in this matter that are to be considered. The most secure option from the perspective of the TSO, is to fix all decisions in the first stage, not allowing to change them afterwards. Inevitably, a stricter policy by the TSO poses additional limits to the suppliers’ flexibility to adjust their capacity and storage planning during the final two stages.

At any stage, the suppliers can purchase entry and exit capacity from the TSO (the *primary* market). These capacities are required to be able to inject or extract gas from the network during the period of operation, which takes place during the final, intraday, stage. When determining whether to purchase entry or exit capacity, suppliers are faced with the choice of purchasing capacity now or waiting until later. During the later stages, the purchasing costs are higher, but more information on prices and demands is known. Furthermore, in the second and third stages, capacity can be traded among suppliers in a *secondary* capacity market, allowing suppliers more freedom to adjust their capacity bookings as they gain more information.

Costs are associated with capacity booking, production, storage, and flow. Maximum flow capacities apply to the pipelines in the network, and gas flows may be subject to losses due to compression. Entry and exit capacities are not explicitly given, but implicitly as a result of feasible flows given the pipeline capacities. Different production capacities apply at each of the suppliers’ production locations. Suppliers are subject to contractual obligations regarding the delivery of specific volumes of gas to certain markets, so-called minimum demands. The gas prices and minimum demands are subject to uncertainty, which impacts supplier behavior.

**Fig. 1:**
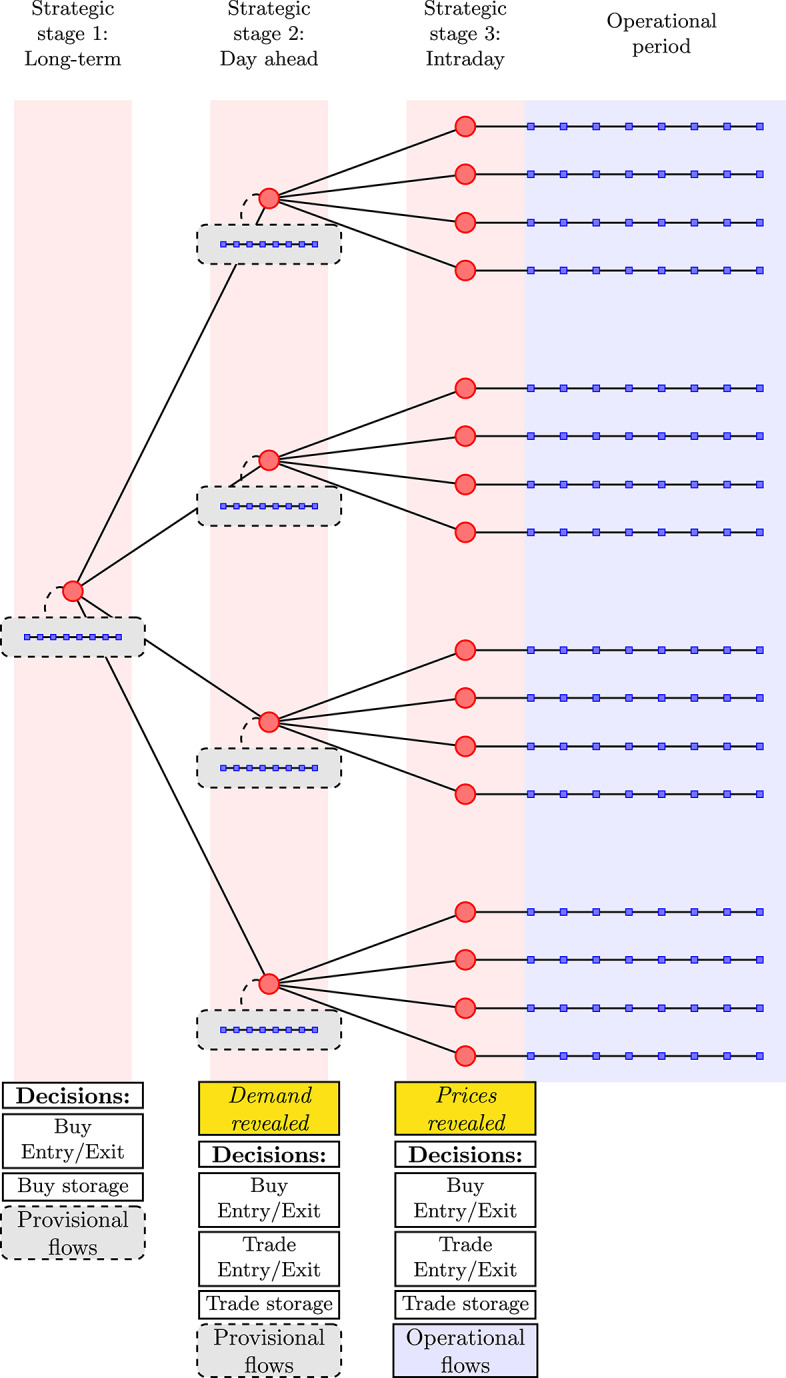
A schematic overview of the strategic planning and operation horizons considered in this study. Entry/exit and storage refers to capacities acquired by suppliers. Provisional correspond to the flows routed by the TSO in the first and second stages, whereas the operational flows are the flows that actually materialize in the third stage

Evidently, demand and prices in markets can fluctuate throughout the day. To address these fluctuations, suppliers have the option to reserve storage capacity at the market nodes against a fee. This option allows them to bring gas to a node, without having to sell immediately. Gas stored in this manner can then be extracted from the storage during a later time block with higher prices, complementing supply extracted directly from the pipeline network. The storage capacity needs to be reserved during the “Long-term” stage, but these capacities can still be traded in a secondary market between different suppliers during the “Day ahead” and “Intraday” stages. A schematic overview of the planning and operation horizons is given in the lower part of Fig. 1, which also presents the realization of uncertain events.

### On secondary market trading

We will now proceed by explaining the dynamics of the entry and exit capacity market followed by a few remarks on the pricing mechanics in the described market context.

#### Illustrative example

**Fig. 2:**
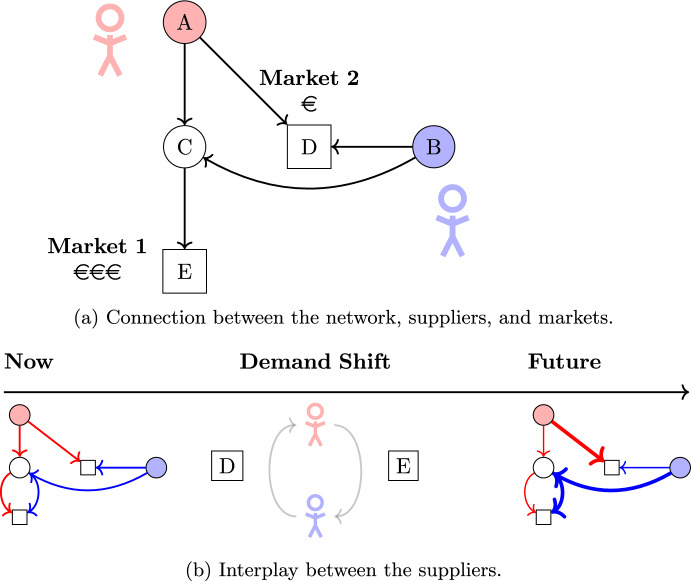
Illustration of the dynamics of the entry and exit capacity markets

To illustrate the dynamics of entry and exit capacity markets in the context of natural gas trading, we present a small example, illustrated by Fig. 2.

We look at a simple network of five nodes and five arcs, as shown in Fig. 2a, where node *C* is an intermediate node. We assume a capacity of ten gas units for each arc. The length of the arcs corresponds to their flow costs; i.e., long arcs are expensive to route gas through. Consider two distinct markets for natural gas: Market 1 and Market 2, represented by nodes *E* and *D* in our network, respectively. The selling prices of natural gas differ between the two; prices are higher in Market 1 and lower in Market 2. Consequently, suppliers prefer to prioritize selling their gas in Market 1 to maximize revenue.

The example involves two suppliers, Supplier 1 and Supplier 2, who operate separate production facilities located at nodes *A* and *B* in our network, respectively, each with a maximum production capacity of ten units of gas. However, contractual obligations require Supplier 1 to deliver at least five units of gas to Market 2 (Node *D*). Despite this obligation, Supplier 1, like Supplier 2, prefers selling gas in Market 1 (Node *E*) due to its more attractive selling prices.

Under the initial scenario, Supplier 1’s contractual minimum of five units for Market 2 leaves them free to allocate the remaining five units of their production to Market 1, which optimally balances Supplier 1’s contractual commitments and revenue goals. As Supplier 1 has lower transportation costs to Market 1 than Supplier 2, the system prioritizes Supplier 1, as stated in the objective of our mathematical model in Eq. (1). Hence, both suppliers sell 5 gas units in Market 1, due to the arc capacity of ten gas units. Consequently, the suppliers book an entry capacity of ten units at node *A* and *B*, respectively, and both book an exit capacity of five units at both nodes *D* and *E*.

Now, consider a shift in conditions: The minimum demand in Market 2 that Supplier 1 must satisfy increases from five units to eight units. An increase is typically allowed within the nomination flexibility of the buyer, see Ason (2022). For illustrative purposes, we make this increase large. This change forces a reallocation of Supplier 1’s supply. With a total production capacity of ten units, fulfilling the additional three units required for Market 2 comes at the expense of Supplier 1’s supply to Market 1.

To adjust to the new situation, Supplier 1 turns to the secondary capacity market. In this market, Supplier 1 purchases three units of exit capacity for Market 2 from Supplier 2 to meet the increased contractual requirement. Simultaneously, Supplier 1 sells three units of exit capacity for Market 1 to Supplier 2 to maintain a balance with their production limit. Supplier 2 is interested in these capacity trades, as they enable a higher profit, due to the higher prices in Market 1. These transactions demonstrate the interplay between contractual obligations, capacity limits, and market price incentives, highlighting the critical role of the secondary market in optimizing entry/exit capacity allocation.

#### Remarks on pricing mechanics

Transactions such as the one we saw in our illustrative example, take place in our model whenever the buying supplier has a higher valuation of the traded capacity than the selling supplier. There can be numerous reasons for this:

1. the selling supplier has an excess of capacity in some market (e.g., because of high contractual obligations needed to be met in other markets); or
2. both suppliers could use the capacity but one supplier can make more profit from obtaining this capacity. In practice, this will only happen when the total capacity already allocated by the TSO is equal to the total capacity that can be allocated in this market: if the TSO still has capacity left to allocate, it is more profitable to buy more capacity directly from the TSO; or
3. the buying supplier needs the capacity to comply with contract feasibility constraints. In reality, this will correspond to a rather high valuation for the buying supplier, as they can expect a large negative financial consequence if they breach their contract.

We remark here that, for any given first (*second*) stage solution, a secondary market transaction will take place between two parties if it increases the second (*third*) stage valuation. The price of these secondary market transactions may differ from the price of obtaining capacity through the TSO, and will depend on both buyer and seller valuations and market circumstances. As the total system profit is always indifferent to the agreed prices for these transactions, there is no need to explicitly consider the price realization as a separate supplier decision. If necessary, one can estimate the prices a posteriori.

### Markets for network and storage capacity

As a starting point for the capacity market model in our context, we consider a situation where the entire system is managed by a single TSO, with its own entry–exit capacity market, inelastic demand aggregated at exit points, own price and contractual demand scenarios, and storage facilities. In reality, market set-ups are much more complex, for instance considering connections to surrounding networks in neighboring countries, numbers and locations of storage facilities, behavior of market segments, etc. We will address some of this complexity relating to market segmentation in our case study. We account for this phenomenon in our methodology in Sect. 4, and illustrate the situation we consider with a small example.

**Fig. 3:**
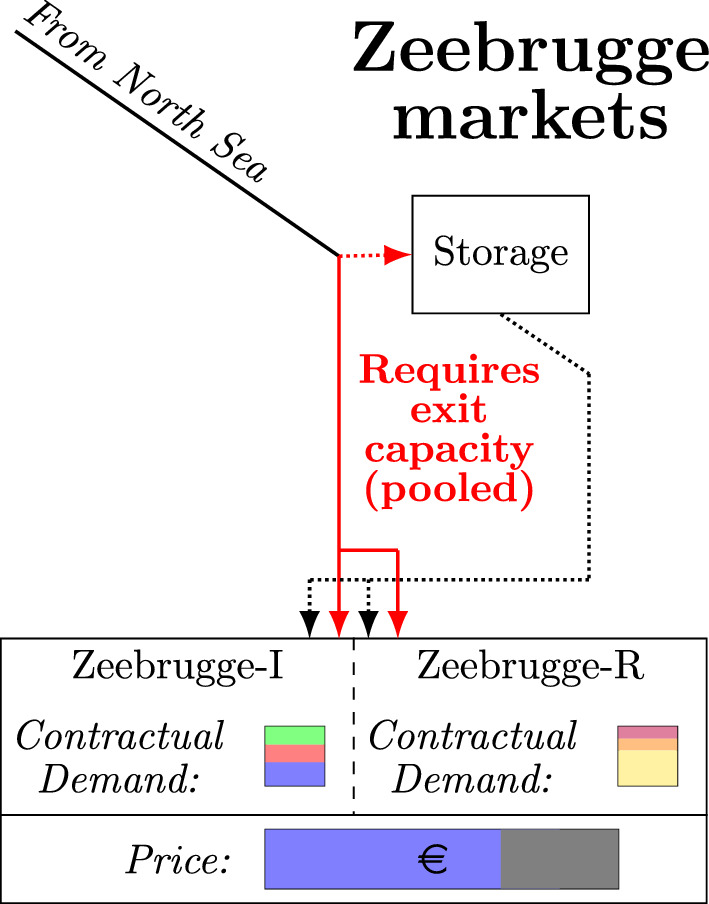
Schematic example of market with submarkets

Figure 3 gives a schematic overview of the situation in the Zeebrugge market in our case study. We can see in this figure that the market consists of two market segments (**I**ndustrial and **R**etail), which pay the same spot prices, but which each have separate contractual agreements with traders. Furthermore, the storage facility and exit capacities in the model at the dummy node can be used to serve either market segment.

## Methodology

In this section, we introduce the complete mathematical formulation of our model. We will first introduce the relevant parameters and variables. Thereafter, we present the mathematical formulation of the stochastic model by stating the objective function and constraints in terms of these variables.

### Scenario tree structure, sets & parameters

To capture the uncertainty inherent to the problem, we employ a stochastic scenario tree with three stages, corresponding to the moments when decisions are taken, as presented in Sect. 3 and shown in Fig. 1. We let $K:= \{1,2,3\}$ be the set of stages and *M* be the set of all strategic nodes in the scenario tree (represented by red circles in Fig. 1). We introduce shorthand notations $M^{(k)}, \; k \in K$ to denote the set of nodes in stage *k*. Furthermore, we introduce $\Pi (m)$ to be the set of all parent scenario nodes of scenario node $m \in M^{(3)}$, including itself. Each strategic node *m* has its corresponding probability $p_m$, such that $\sum _{m\in M^{(k)}} p_m = 1$ for $k \in K$ (probabilities within a stage sum to 1), and $p_{m} = \sum _{\tilde{m} \in M^{(3)}: m \in \Pi (\tilde{m})} p_{\tilde{m}}$ for all $m \in M^{(k)}$, $k \in K {\setminus } \{3\}$ (probability of a parent node is the sum of the probabilities of its children in the third stage). We let the set *H* describe the different time blocks in which the operational period is partitioned, such that each operational node (represented by blue squares in Fig. 1) is indexed on both *M* and *H*. The set *T* represents different suppliers in our model. Finally, the physical infrastructure is modeled as a directed graph $G=(N,A)$, where *N* represents nodes (facilities or markets) and *A* represents arcs (pipelines).

Gas flow in the network is subject to losses due to compression, represented by a loss rate $l_{a}$ for each arc $a \in A$. Costs associated with capacity booking, production, storage, and flow, are represented by $c^{N+}_{mh}$ (entry), $c^{N-}_{mh}$ (exit), $c^{P}_{n}$, $c^{I}_{n}$, and $c^{A}_{a}$, respectively. Each arc *a* has a maximum flow capacity $CAP^{A}_{a}$, and each node *n* has a maximum production capacity $CAP^{P}_{n}$.

The prices and demands volumes are subject to uncertainty, modeled through uncertain parameters $\xi _{nmht}$ for demand and $r_{nmh}$ for price. The parameters $C_1$ and $C_2$ are used to reflect the level of the TSO’s risk aversion and limit respective changes in flow, capacity and storage planning after the first and second stage decisions have been made.

### Variables

The model contains several non-negative decision variables: $x^{+}_{nmht}$ and $x^{-}_{nmht}$ respectively denote the entry and exit capacity at node *n* acquired by supplier *t* in scenario node *m*. Variables $y^{+}_{nmht}$ and $y^{-}_{nmht}$ respectively represent the entry and exit capacities sold by suppliers in the secondary market. Variables $s^{+}_{nmh}$ and $s^{-}_{nmh}$ denote the capacity sold by the TSO. Gas flow for a supplier at arc *a* in scenario node *m* is denoted by $f_{mhat}$, while $q^{S}_{nmht}$ and $q^{P}_{nmht}$ represent the quantity sold and produced, respectively. Storage is modeled using $v_{nmht}$, $w^{+}_{nmht}$, and $w^{-}_{nmht}$, representing the stored inventory, the gas volume injected into storage, and the gas volume retrieved from storage, respectively. We model buying and selling storage capacity with decision variables $z_{nmht}$ and $u_{nmht}$, respectively.

For a comprehensive overview of all sets, parameters, and decision variables, please refer to Tables 8, 9, and 10 in Appendix 1.

### Multi-stage stochastic program

***Objective***

1 $$\begin{aligned}\max _{\begin{array}{c} \boldsymbol{x}, \boldsymbol{y}, \boldsymbol{s}, \boldsymbol{q}, \\ \boldsymbol{f}, \boldsymbol{v}, \boldsymbol{w} \end{array}} &\quad \sum _{n \in N} \sum _{t \in T} \sum _{h \in H} \left[ \sum _{m \in M^{(3)}} p_{m} \cdot \underbrace{q^{S}_{nmht} \cdot r_{nmh}}_{\text {Supplier sales revenue}} \right.\\ &\quad -\sum _{m \in M^{(3)}} p_{m} \cdot \left( \underbrace{c^{P}_{n} \left( q^{P}_{nmht} \right) }_{\text {Supplier production costs}} {+} \sum _{a \in A^{+}_{n}}\underbrace{c^{A}_{a} \left( f_{mhat} \right) }_{\text {Flow costs}} \right)\\ & \quad -\sum _{m \in M} p_{m} \cdot \left( \underbrace{c^{N+}_{mh} \left( x^{+}_{nmht} - y^{+}_{nmht} \right) }_{\text {Costs entry capacity}} + \underbrace{c^{N-}_{mh} \left( x^{-}_{nmht} - y^{-}_{nmht} \right) }_{\text {Costs exit capacity}} \right.\\ &\quad +\left. \underbrace{c^{I}_{nmh} \left( z_{nmht} - u_{nmht} \right) }_{\text {Supplier costs storage capacity}} \right)\\ &\quad -\sum _{m\in M}p_m\cdot \left( \underbrace{\epsilon \left( w^{-}_{nmht} + v_{nmht} \right) }_{\text {Penalty for storage use and extraction}} \right.\\ & \quad \left. \left. +\underbrace{\epsilon \cdot \left( y^{+}_{nmht}+ y^{-}_{nmht} + u_{nmht} \right) }_{\text {Penalty for sales}} \right) \right]\end{aligned}$$

The objective of the TSO is to operate the network under a transportation plan that maximizes social welfare, while taking into account the flow costs associated with transporting gas. The social welfare maximization is driven by suppliers competing for capacity aiming to supply their gas to the most profitable markets. In reality, prices of capacity usage are charged to the suppliers (regulated, or auction-determined). In our objective, charges paid from suppliers to the TSO will cancel out (Egging 2010, Footnote 203). However, actual costs to cover operations, maintenance, depreciate, overhead etc., are reflected in the objective function. Under the assumptions we have made, this objective leads to a maximized social welfare (Egging 2010, Page 72). We penalize sales, storage use and extraction with an $\epsilon$ to prevent degenerate solutions.

***TSO constraints***

2a $$\begin{aligned}&\sum _{t \in T} f_{mhat} \le CAP^{A}_{a} \qquad&\forall m \in M, h \in H, a \in A \end{aligned}$$

Constraint (2a) ensures that the arc capacities are not exceeded.

***Supplier constraints***

2b $$\begin{aligned} q^{S}_{nmht} \ge \xi _{nmht}&\qquad \forall n \in N, m \in M^{(3)}, h \in H, t \in T \end{aligned}$$

2c $$\begin{aligned} \sum _{t \in T} q^{P}_{nmht} \le CAP^{P}_{n}&\qquad \forall n \in N, m \in M^{(3)}, h \in H \end{aligned}$$

2d $$\begin{aligned}&\begin{aligned}&q^{P}_{nmht} + w^{-}_{nmht} + \sum _{a \in A^{+}_{n}} \left( 1 - l_{a} \right) f_{mhat} = q^{S}_{nmht} + w^{+}_{nmht} + \sum _{a \in A^{-}_{n}} f_{mhat}\\&\quad \forall n \in N, m \in M^{(3)}, h \in H, t \in T \end{aligned} \end{aligned}$$

2e $$\begin{aligned}&\begin{aligned} \sum _{\tilde{m} \in \Pi (m)} \left( x^{-}_{n \tilde{m} h t} - y^{-}_{n \tilde{m} h t} \right) \ge q^{S}_{nmht} + w^+_{nmht} - w^-_{nmht}\\\forall n \in N, m \in M^{(3)}, h \in H, t \in T \end{aligned} \end{aligned}$$

2f $$\begin{aligned} \sum _{\tilde{m} \in \Pi (m)} \left( x^{+}_{n \tilde{m} h t} - y^{+}_{n \tilde{m} h t} \right) \ge q^{P}_{nmht}&\qquad \forall n \in N, m \in M^{(3)}, h \in H, t \in T \end{aligned}$$

2g $$\begin{aligned} v_{nmht} = \sum _{\tilde{h} = 1}^{h} \left( w^{+}_{n m \tilde{h} t} - w^{-}_{n m \tilde{h} t} \right)&\qquad \forall n \in N, m \in M^{(3)}, h \in H, t \in T \end{aligned}$$

2h $$\begin{aligned} v_{nmht} \le CAP^{I}_{n}&\qquad \forall n \in N, m \in M^{(3)}, h \in H, t \in T \end{aligned}$$

2i $$\begin{aligned} v_{nmht} \le \sum _{\tilde{m} \in \Pi (m)} \left( z_{n\tilde{m}ht}-u_{n\tilde{m}ht} \right)&\qquad \forall n \in N, m \in M^{(3)}, h \in H, t \in T \end{aligned}$$

In constraint (2b), we ensure that the nominated contract demand is met by the supplier’s sales. Constraint (2c) ensures that the suppliers do not produce more than the capacity at the node. Constraint (2d) entails the mass-balance constraints. We ensure that the production, extraction from storage, and loss-corrected inflows equal the sum of sales, injection into storage, and outflows. We adjust the inflows by a factor $(1-l_a)$ since compressors maintaining pressure in the network use a fraction of the transported gas. Constraint (2e) accounts for the bought and sold exit capacity: Exit capacity is needed for all sales that are realized by taking gas directly from the network, as well as for injection into storage, but not for sales that are realized using stored gas (which has already exited the system). Similarly, Constraint (2f) ensures that suppliers have enough entry capacity to feed in their production. Constraint (2g) models the storage of gas and extraction/injection out of/into the storage. We model the storage inventory capacity limit with Constraint (2h). The storage inventory is limited by the total net purchased storage capacity over all stages, as indicated by Constraint (2i) models the storage of gas and extraction/injection out of/into the storage. Note that $v_{nmht}$ is non-negative, and it will never be optimal to have positive inventory after the last time block, so we do not need to explicitly account for an injections-extractions balance. From a storage perspective, injection and withdrawal limits may apply in reality. We assume that upon purchasing storage capacity, a complementary amount of injection and withdrawal capacity is included. These offered capacities are assumed to be sufficiently high, such that additional cost for injecting and withdrawing from the storage does not have to be considered explicitly.

***Storage booking constraints***

2j $$\begin{aligned}&z_{nmht} = z_{nm1t} \quad&\forall n \in N, m \in M, h \in H, t\in T \end{aligned}$$

2k $$\begin{aligned}&u_{nmht} = u_{nm1t} \quad&\forall n \in N, m \in M, h \in H, t\in T \end{aligned}$$

Constraints (2j) and (2k) ensure that the storage capacity that is bought is equal throughout the entire day. This enforces the most risk-averse capacity management approach. Relaxing these constraints allows the purchased storage capacity to be different for each time block, increasing flexibility.

***Capacity market constraints***

2l $$\begin{aligned}&\sum _{t \in T} x^{-}_{nmht} = s^{-}_{nmh} + \sum _{t \in T} y^{-}_{nmht} \quad&\forall n \in N, m \in M, h \in H \end{aligned}$$

2m $$\begin{aligned}&\sum _{t \in T} x^{+}_{nmht} = s^{+}_{nmh} + \sum _{t \in T} y^{+}_{nmht} \quad&\forall n \in N, m \in M, h \in H \end{aligned}$$

2n $$\begin{aligned}&\sum _{t \in T} z_{nmht} = \sum _{t \in T} u_{nmht} \quad&\forall n \in N, m \in \bigcup _{k \in K \setminus \{1\}} M^{(k)}, h \in H \end{aligned}$$

Constraints (2l) and (2m) balance the selling and buying of the secondary market exit and entry capacities. In every scenario node, the entry and exit capacity volume bought by the suppliers equals the volume sold by the TSO and the suppliers in the secondary market. Similarly, Constraint (2n) ensures that storage capacity in stages 2 and 3 can only be purchased through the secondary market, by equating the total storage capacity purchased by suppliers to the total storage capacity sold by suppliers. This is different from entry–exit trading, for which capacity can be purchased from the TSO also in stages 2 and 3.

***Risk aversion constraints***

Although the TSO is risk neutral with regard to the system social welfare objective (Eq. (1)), there are some risks inherent to allowing for capacity adjustments shortly before operation. Viewed through the lense of a TSO engineer, too big of a last-minute change in capacity and flow planning may jeopardize the guarantee of being able to stably operate a transportation plan that suits the suppliers’ bids. In the worst case, this leads to the TSO not meeting its own obligations, which it wants to avoid at all costs. These risks are not reflected in Eq. (1), or any of the constraints introduced before. We will introduce constraints to model the conservative stance of the TSO in this matter, and show that these emerge from applying chance constraints to (a slightly adjusted version of) our problem. Furthermore, in Appendix 2, we will argue that, under specific assumptions, applying chance constraints also constrains the VaR and CVaR of an adjusted objective, due to an existing equivalence relation between chance constraints and VaR (Sarykalin et al. 2008).

Let us assume that the occurrence of a failure of meeting obligations depends on a controllable variable $\eta$, and an uncertain variable $\xi$. More precisely, let us say that a failure occurs when $\phi (\eta ,\xi )> \tau$, where $\phi$ is a function that is non-decreasing in $\eta$.

Logically, the TSO aims to constrain the risk of a failure to an acceptable, very low probability. At the same time, the TSO would like to retain some degree of flexibility for the gas suppliers to make adjustments to their bids in later stages.

Constraining the failure risk can be achieved by adding a chance constraint to the original model, which ensures that $\mathbb {P} \left[ \phi (\eta , \xi ) \le \tau \right] \ge \alpha$.

As we assumed that $\phi$ is non-decreasing in $\eta$, it holds that $\mathbb {P}[\phi (\eta , \xi )> \tau ]$ is also non-decreasing in $\eta$. This implies that (for values of $\alpha$ that are not extremely low) we can write this single chance constraint as a regular constraint depending on $\alpha$ instead: $\eta \le C(\alpha ,\tau )$. Note that while it may be very hard to compute $C(\alpha ,\tau )$, we know that $C(\alpha ,\tau )$ is non-increasing in $\alpha$.

As established before, failure risk for the TSO increases as the (relative) changes in the flow and capacity plan at later stages w.r.t. earlier stages become larger. Based on our earlier analysis, mitigating failure risk can thus be modeled by imposing a constraint on these changes, acting in the same manner as the constraint on $\eta$ introduced before. The implicit assumption here is that the occurence of a failure depends solely on the relative change in the transportation plan and some unobserved uncertainty. The level of risk the TSO is willing to take increases as the right-hand side of such a constraint increases. To model this, three sets of auxiliary decision variables are used: $\gamma _{imhat}$, $\delta ^{s}_{inmht}$, and $\theta _{inmht}$. The former computes the difference in flow decisions between the third stage and the corresponding first (${k}=1$) or second stage (${k}=2$) decision, shown in Constraint (2q) and (2r). The second measures the difference in acquired entry and exit capacity, [Constraint(2s) and (2t)], and the third measures difference in acquired storage capacity [Constraint(2u) and (2v)]. In these constraints, we make use of some additional auxiliary decision variables: $\lambda ^{s}_{nmht}$ and $\kappa _{nmht}$. $\lambda ^{s}_{nmht}$, keeps track of how much exit ($s=-$) and entry ($s=+$) capacity at node *n* a supplier *t* has acquired so far up to scenario node *m* at time block *h*, as defined in Constraint (2o). $\kappa _{nmht}$, defined in Constraint (2p), tracks the total storage capacity acquired upto node *m* for each node *n* and supplier *t* in a similar fashion. As we want to quantify the absolute deviation from the third stage decisions, we require tracking both positive differences ($\hat{\gamma }$, $\hat{\delta }$, $\hat{\theta }$) and negative differences ($\bar{\gamma }$, $\bar{\delta }$, $\bar{\theta }$) separately.

2o $$\begin{aligned}&\lambda ^{s}_{nmht} = \sum _{\tilde{m} \in \Pi (m)} \left( x^{s}_{n \tilde{m} h t} - y^{s}_{n \tilde{m} h t} \right)&\left\{ \begin{array}{ll} \forall s \in \{-, +\}, n \in N, \\ m \in M, h \in H, t \in T \end{array} \right. \end{aligned}$$

2p $$\begin{aligned}&\kappa _{nmht} = \sum _{\tilde{m} \in \Pi (m)} \left( z_{n \tilde{m} h t} - u_{n \tilde{m} h t} \right)&\forall n \in N, m \in M, h \in H, t \in T \end{aligned}$$

2q $$\begin{aligned}&\hat{\gamma }_{{k}mhat} \ge f_{mhat} - f_{\tilde{m}hat} \quad&\left\{ \begin{array}{ll} \forall m \in M^{(3)}, \tilde{m} \in \Pi (m),\\ h \in H, a \in A, t \in T;\; {k}: \tilde{m} \in M^{({k})} \end{array} \right. \end{aligned}$$

2r $$\begin{aligned}&\bar{\gamma }_{{k}mhat} \ge f_{\tilde{m}hat} - f_{mhat} \quad&\left\{ \begin{array}{ll} \forall m \in M^{(3)}, \tilde{m} \in \Pi (m),\\ h \in H, a \in A, t \in T;\; {k}: \tilde{m} \in M^{({k})} \end{array} \right. \end{aligned}$$

2s $$\begin{aligned}&\hat{\delta }^{s}_{{k}nmht} \ge \lambda ^{s}_{nmht} - \lambda ^{s}_{n\tilde{m}ht} \quad&\left\{ \begin{array}{ll} \forall s \in \{-, +\}, n \in N, m \in M^{(3)}, \\ \tilde{m} \in \Pi (m), h \in H, t \in T;\;{k}: \tilde{m}\in M^{({k})} \end{array} \right. \end{aligned}$$

2t $$\begin{aligned}&\bar{\delta }^{s}_{{k}nmht} \ge \lambda ^{s}_{n\tilde{m}ht} - \lambda ^{s}_{nmht} \quad&\left\{ \begin{array}{ll} \forall s \in \{-, +\}, n \in N, m \in M^{(3)},\\ \tilde{m} \in \Pi (m), h \in H, t \in T;\; {k}: \tilde{m}\in M^{({k})} \end{array} \right. \end{aligned}$$

2u $$\begin{aligned}&\hat{\theta }_{{k}nmht} \ge \kappa _{nmht} - \kappa _{n\tilde{m}ht} \quad&\left\{ \begin{array}{ll} \forall n \in N, m \in M^{(3)}, \tilde{m} \in \Pi (m),\\ h \in H, t \in T;\; {k}: \tilde{m} \in M^{({k})} \end{array} \right. \end{aligned}$$

2v $$\begin{aligned}&\bar{\theta }_{{k}nmht} \ge \kappa _{n\tilde{m}ht} - \kappa _{nmht} \quad&\left\{ \begin{array}{ll} \forall n \in N, m \in M^{(3)}, \tilde{m} \in \Pi (m),\\ h \in H, t \in T;\; {k}: \tilde{m} \in M^{({k})} \end{array} \right. \end{aligned}$$

In the below, parameters $C_1$ and $C_2$ are used to quantify the TSO’s risk aversion. The former quantifies the allowed maximum total difference in decisions between the first and the third stage whereas the latter does the same for the second and the third stage decisions. These values correspond to the levels of risk $\alpha _1$ and $\alpha _2$ the TSO is willing to take w.r.t a failure to meet obligations. Both correspondences are monotonous, i.e. a higher risk tolerance $1-\alpha _{{k}}$ corresponds to a higher $C_{{k}}$, but the exact relation of $C_1$ to $\alpha _1$ may differ from that of $C_2$ to $\alpha _2$. By definition, we have $C_2 \le C_1$. We constrain the sum of absolute changes in decision variables by the sum over all the corresponding third stage decision variables, multiplied with the respective risk-aversion parameter $C_{{k}}$ in Constraints (2w), (2x) and (2y). As a general remark, a relatively high value of $C_{{k}}$, e.g. an aggregated deviation of 50% for the final stage with respect to the planning at stage *k*, may still correspond to an acceptable, extremely low risk tolerance.

2w $$\begin{aligned}&\sum _{n\in N}\sum _{t \in T} \left( \hat{\delta }_{{k}nmht} + \bar{\delta }_{{k}nmht}\right) \le C_{{k}} \sum _{n \in N} \sum _{t \in T} \lambda ^s_{n\tilde{m}ht}&\left\{ \begin{array}{ll} \forall s \in \{-, +\}, m \in M^{(3)},\\ \tilde{m} \in \Pi (m), h \in H;\; {k}: \tilde{m}\in M^{({k})} \end{array} \right. \end{aligned}$$

2x $$\begin{aligned}&\sum _{a\in A}\sum _{t \in T} \left( \hat{\gamma }_{{k}mhat} + \bar{\gamma }_{{k}mhat}\right) \le C_{{k}} \sum _{a \in A} \sum _{t \in T} f_{\tilde{m}hat}&\left\{ \begin{array}{ll} \forall m \in M^{(3)},\tilde{m} \in \Pi (m),\\ h \in H;\; {k}: \tilde{m}\in M^{({k})} \end{array} \right. \end{aligned}$$

2y $$\begin{aligned}&\sum _{n\in N}\sum _{t \in T} \left( \hat{\theta }_{{k}nmht} + \bar{\theta }_{{k}nmht}\right) \le C_{{k}} \sum _{n \in N} \sum _{t \in T} \kappa _{n\tilde{m}ht}&\left\{ \begin{array}{ll} \forall m \in M^{(3)},\tilde{m} \in \Pi (m),\\ h \in H;\; {k}{:}\, \tilde{m}\in M^{({k})} \end{array} \right. \end{aligned}$$

***Submarket constraints***

We model the submarket setting outlined in Sect. 3.2 using three market nodes: one dummy market node used for accounting the shared exit capacity and storage, and two end nodes for the respective market segments R and I, that can be reached from the dummy node using an arc with zero flow cost. As sales should take place in the market segments and not in the dummy node, we set the sales price at the dummy node to €0. Let *S*(*n*) be the set of market segments corresponding to a (dummy) node *n* in this fashion (Note: for almost all network nodes *n*, we have $S(n) = \emptyset$). As the exit capacities at the market segment share the total pooled exit capacity purchased in the dummy node, we cap these exit capacities by enforcing the following constraints:

2z $$\begin{aligned} \begin{aligned} \sum _{\tilde{n} \in S(n)}\sum _{\tilde{m} \in \Pi (m)} \left( x^{-}_{\tilde{n} \tilde{m} h t} - y^{-}_{\tilde{n} \tilde{m} h t} \right) - w^-_{nmht} + w^+_{nmht} \le \sum _{\tilde{m} \in \Pi (m)} \left( x^{-}_{n \tilde{m} h t} - y^{-}_{n \tilde{m} h t} \right) \\\forall n \in N, m \in M^{(3)}, h \in H, t \in T \end{aligned} \end{aligned}$$

As Constraint (2z) ties exit capacities for the market segment nodes to the exit capacity purchased at the dummy node, the cost for obtaining exit capacity at the market segment nodes themselves is set to 0. The terms $- w^-_{nmht} + w^+_{nmht}$ in Constraint (2z) ensure that gas extracted from the pooled storage does not use exit capacity, whereas injecting into the pooled storage does. Finally, we remark that Constraint (2e) is valid at both the dummy node and the market segment nodes, albeit partially redundant.

***Domain constraints***

$$\begin{aligned}&f_{mhat} \in \mathbb {R}^{+} \quad&\forall m \in M, h \in H, a \in A, t \in T \\&\left. \begin{array}{l} x^{+}_{nmht}, \quad x^{-}_{nmht}, \\ y^{+}_{nmht}, \quad y^{-}_{nmht} \end{array} \right\} \in \mathbb {R}^{+} \quad&\forall n \in N, m \in M, h \in H, t \in T \\&s^{+}_{nmh}, \quad s^{-}_{nmh} \in \mathbb {R}^{+} \quad&\forall n \in N, m \in M, h \in H \\&\left. \begin{array}{l} q^{P}_{nmht}, \quad v_{nmht}, \\ w^{+}_{nmht}, \quad w^{-}_{nmht} \end{array} \right\} \in \mathbb {R}^{+} \quad&\forall n \in N, m \in M^{(3)}, h \in H, t \in T \\&q^{S}_{nmht} \in \mathbb {R}^{+} \qquad&\forall n \in N, m \in \bigcup _{k \in K \setminus \{1\}} M^{(k)}, h \in H, t \in T \\&\lambda ^{s}_{nmht} \in \mathbb {R}^{+} \qquad&\forall s \in \{-,+\}, n \in N, m \in M, h \in H, t \in T \\&\kappa _{nmht}\in \mathbb {R}^{+} \qquad&\forall n \in N, m \in M, h \in H, t \in T \\&\hat{\gamma }_{kmhat}, \bar{\gamma }_{kmhat} \in \mathbb {R}^{+} \qquad&\forall k \in K \setminus \{3\}, m \in M^{(3)}, h \in H, a \in A, t \in T \\&\hat{\delta }^{s}_{knmht}, \bar{\delta }^{s}_{knmht} \in \mathbb {R}^{+}&\left\{ \begin{array}{l} \forall s \in \{-,+\}, k \in K \setminus \{3\}, \\ n \in N, m \in M^{(3)}, h \in H, t \in T \end{array} \right. \\&\hat{\theta }_{knmht}, \bar{\theta }_{knmht} \in \mathbb {R}^{+}&\forall k \in K \setminus \{3\}, n \in N, m \in M^{(3)}, h \in H, t \in T \\&z_{nmht}, \quad u_{nmht} \in \mathbb {R}^{+} \quad&\forall n \in N, m \in M, h \in H, t \in T \end{aligned}$$

All decision variables lie in the domain of non-negative real numbers. Note that we set the upper bound of production and sales decision variables to zero if the supplier cannot produce respectively trade on that node. Additionally, we set the upper bound of the decision variables corresponding to buying entry capacity at a node equal to zero if a supplier is not active at that node.

## Results and discussion

To study ways of optimizing the gas entry–exit capacity utilization under uncertainty, we describe the experimental setup in Sect. 5.1 and discuss the numerical results of the case study in Sect. 5.2.

### Experimental setup

We explain the data used in this case study in three parts: network and supplier data, parameter values, and scenarios. Next, we restate the research questions and explain how we answer them.

#### Network and supplier data

**Fig. 4:**
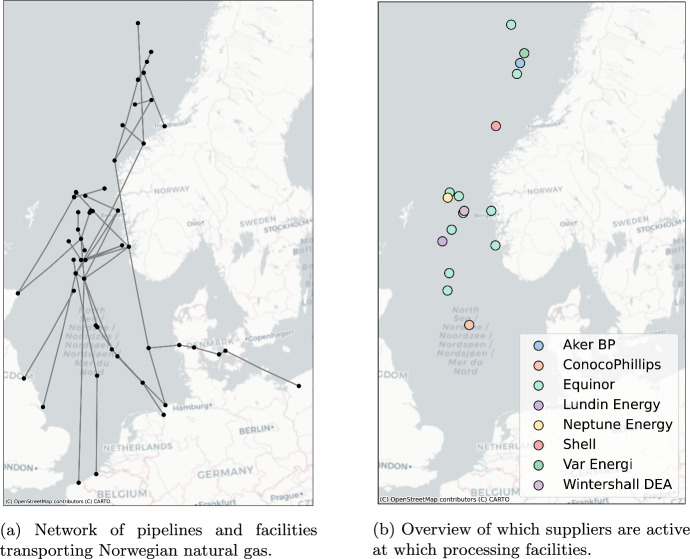
Network and supplier data used in this study

In our study, we utilize a comprehensive dataset concerning the gas pipeline network, sourced primarily from GeoNorge (2024). This dataset provides detailed information about the locations of pipelines and facilities, as well as their capacities. As this dataset pertains to a subset of all the pipelines in this area, we have augmented the dataset with additional pipelines based on information from Norsk Petroleum (2024), such as those connecting Denmark to Poland. The pipeline capacities reported by GeoNorge (2024) were verified against the capacities displayed in Gassco (2024). The resulting network, consisting of 65 nodes and 67 pipe lines, is visualized in Fig. 4a. We divide the nodes into three categories: (1) facilities for accumulating production and/or processing; (2) intermediate nodes; and (3) markets. Gas is fed into the network at the processing nodes, and passes through the intermediate nodes to end up in the markets.

We have obtained production capacity data from GEM Wiki (2024), for which we mapped each production node to a facility in our network, based on information about the connecting pipelines. If this data was missing, we made a connection to the nearest facility in our network. Additionally, we used information from this source to determine which suppliers are active at specific facilities or nodes, and which act as the main operators of processing facilities.

We consider eight different suppliers, each of them only being active at only a subset of the processing facilities, as shown in Fig. 4b. In Figs. 12 and 13 in “Appendix 3”, we visualize the network range of the different suppliers.

#### Parameter values

In this section, we discuss the parameter values for our case study. We will first motivate our choices for fixed parameters, such as booking and flow costs. This is followed by an explanation of the parameters that are affected by uncertainty.

***Fixed parameters***

For the fixed parameter values, we have made several deliberate choices to construct realistic economic dynamics. We want to incentivize early booking of capacity that will definitely be used. Additionally, we want to enable booking capacity that is only used circumstantially at a later stage. Therefore, the costs for buying and selling entry and exit capacities in each of the three stages were set to €1, €1.04, and €1.08 per MWh, respectively, based on information from Gassco (2025). Preliminary results showed that this yields a balanced distribution of trading over the stages. The flow costs were set uniformly to €0.10 per MWh for traversing each pipeline, as we do not want flow cost in itself to be a main driver of the results. Storage costs were set to €2.40 for storage capacity of one MWh for 24 hours, which is equivalent to a cost of €0.30 per three-hour period. We consider the former an appropriate value as it makes the use of storage profitable in case of considerable price fluctuations over time, yet not in case of minor fluctuations. Production costs amount to €9 per MWh, which is approximately 30% of the gas price per MWh, similar to relative costs reported in Equinor (2024). We assume that the storage facilities can accommodate all capacity requests, i.e., there are no hard capacity limits. We neglect losses in the gas flow, which is equivalent to a loss rate of 0%. An overview of all the parameters and their values can be found in Table 2.

**Table 2:** Values for fixed parameters

Description	Parameter	Domain	Value	Unit
Entry and exit capacity costs	$c^{N+}_{mh}, c^{N-}_{mh}$	$\forall m \in M^{(1)}, h \in H$	1	€/MWh
	$c^{N+}_{mh}, c^{N-}_{mh}$	$\forall m \in M^{(2)}, h \in H$	1.04	
	$c^{N+}_{mh}, c^{N-}_{mh}$	$\forall m \in M^{(3)}, h \in H$	1.08	
Production costs	$c^{P}_{n}$	$\forall n \in N$	9	€/MWh
Storage cap. costs	$c^{I}_{n}$	$\forall n \in N$	0.3	€/MWh per time block
Flow costs	$c^{A}_{a}$	Market $\rightarrow$ Segments	0	€/MWh
		All other arcs	0.1	€/MWh
Production capacity	$CAP^{P}_{n}$	$\forall n \in N$	Depends on *n*	MWh per time block
Pipe capacity	$CAP^{A}_{a}$	$\forall a \in A$	Depends on *a*	MWh per time block
Storage cap. limit	$CAP^{I}_{n}$	Market nodes	$\infty$	MWh
		All other nodes	0	MWh
Loss rate	$l_{a}$	$\forall a \in A$	0	Not applicable

***Parameters affected by uncertainty***

**Fig. 5:**
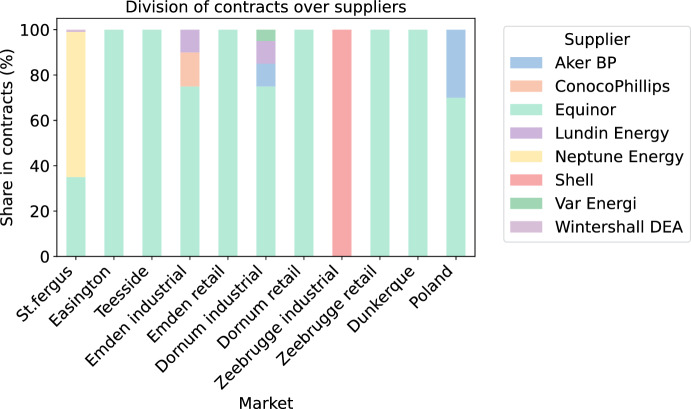
Minimum contract volumes in each market for each supplier

The uncertain minimum demand contract volumes for gas and the uncertain gas prices for each (sub)market are provided in Tables 3 and 4, respectively. These values were chosen to be aligned with real values for the specific (sub)markets (Intercontinental Exchange 2025; Gassco 2024). We divide a day of 24 h into eight time blocks (TB1 up to TB8) of 3 h each. To mimic a realistic daily cycle of demand levels over the day, we group these time blocks such that we have four periods (P1 up to P4) of 12, 3, 6, and 3 h, respectively. We divide the minimum volume contracts in a feasible and fair manner over the different suppliers as shown in Fig. 5.

**Table 3:** Total contractual demands (MWh) for natural gas in different (sub)markets for each three hour time block

Market	Segment	Demand level	P1				P2	P3		P4
			TB1	TB2	TB3	TB4	TB5	TB6	TB7	TB8
St. Fergus	–	–	12,500				12,500	12,500		12,500
Easington	–	–	12,500				12,500	12,500		12,500
Teesside	–	–	12,500				12,500	12,500		12,500
Poland	–	–	25,000				25,000	25,000		25,000
Dunkerque	–	–	50,000				56,250	43,750		50,000
Emden	Industrial	High	25,000				33,125	25,000		33,125
		Low	25,000				23,125	25,000		23,125
	Retail	High	25,000				33,125	25,000		33,125
		Low	25,000				23,125	25,000		23,125
Dornum	Industrial	High	25,000				33,125	25,000		33,125
		Low	25,000				23,125	25,000		23,125
	Retail	High	25,000				33,125	25,000		33,125
		Low	25,000				23,125	25,000		23,125
Zeebrugge	Industrial	High	25,000				35,000	25,000		35,000
		Low	25,000				15,000	25,000		15,000
	Retail	High	25,000				35,000	25,000		35,000
		Low	25,000				15,000	25,000		15,000

We denote the four periods by P1 up to P4 and the eight time blocks by TB1 up to TB8. The combination of *Market* and *Segment* refers to a specific (sub)market. *Demand Level* distinguishes between the low and high demand cases at such a market

**Table 4:** Prices for natural gas in different markets (€/MWh)

Market	Segment	Price level	P1				P2	P3		P4
			TB1	TB2	TB3	TB4	TB5	TB6	TB7	TB8
St. Fergus	–	–	26				28	27		29
Easington	–	–	26				28	27		29
Teesside	–	–	26				28	27		29
Zeebrugge	Both	–	29				31	30		32
Poland	–	–	30				30	30		30
Emden	Both	High	31				30	32		31
		Low	25				30	26		31
Dornum	Both	High	31				30	32		31
		Low	25				30	26		31
Dunkerque	–	High	32				32	33		33
		Low	28				32	29		33

We denote the four periods by P1 up to P4 and the eight time blocks by TB1 up to TB8. The combination of *Market* and *Segment* refers to a specific (sub)market. *Price Level* distinguishes between the low and high price cases at such a market

#### Scenarios

**Fig. 6:**
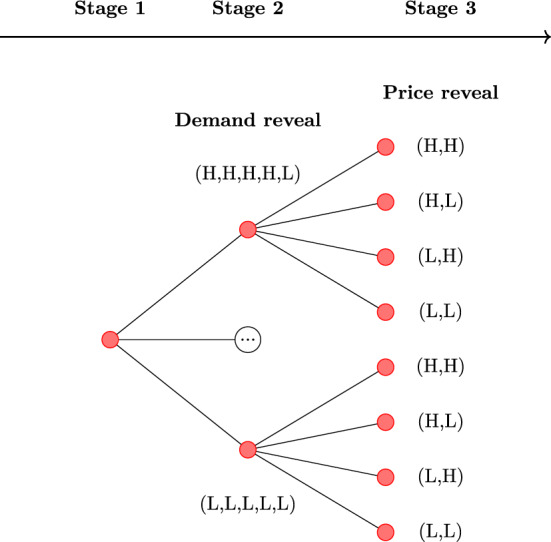
Realization of some of the uncertainties in the schematic scenario tree, including a selection of 8 out of 96 scenarios

In our case study, we consider 96 equiprobable scenarios, which correspond to different realizations of the uncertain minimum demand and the gas prices. Contract nominations are revealed in the second stage, and prices in the third stage, respectively, as visualized in Fig. 6. More specifically, we consider fluctuations in the demand at the market segments in Emden, Dornum and Zeebrugge during time blocks 5 and 8. In these time blocks, demand can be either high (H) or low (L). Most of these fluctuations are assumed to be independent, with two notable exceptions. Firstly, because of the proximity of the Emden and Dornum nodes, the realizations of retail market demand are assumed to be identical in both locations. In addition to this, we model the effect of large fluctuations between different demand realizations in the Zeebrugge market segments. The consequence of this is that high demand cannot be realized in both Zeebrugge market segments simultaneously. In practice, this means that the Zeebrugge market has 3 equiprobable realizations for industrial and retail demand: (H, L), (L, H) and (L, L). This results in $2^3 * 3 = 24$ branches in the second stage, as shown in Fig. 6. Specific values of the demand are provided in Table 3. For the uncertain prices, we consider two possible realizations, high (H) and low (L), at the markets in France and Germany, as shown in Table 4 and Fig. 6. Again, the price realizations in the German markets of Emden and Dornum are assumed to be identical due to geographical proximity, resulting in $2^2 = 4$ different realizations of prices throughout the network.

#### Research questions

In this research, the goal is to study ways of optimizing the gas entry–exit capacity utilization under uncertainty. To achieve this, we conduct experiments that contribute to the resolution of the research questions, as introduced in Sect. 1:

1. How does risk aversion affect capacity allocation and capacity availability in various strategic and operation stages? (Sect. 5.2.1)
2. What are the bottlenecks in the system and how can we solve them? (Sect. 5.2.2)
3. What is the value of booking-flexibility for storage facilities? (Sect. 5.2.3)

For the first research question, we vary the values for $C_1$ and $C_2$. To answer the second and third research questions, we follow the experimental setup as described in Sects. 5.1.1–5.1.3.

#### Computational setup

We have implemented our methods and experiments in Python 3.10, solved the models with the Gurobi 10.0.1 solver (Gurobi Optimization 2025), and published the corresponding data and code on GitHub (Markhorst 2025). For Gurobi, we use an aggresive presolve method and the barrier method, while the other parameters are set to the default values. All experiments were run on a high performance computing (HPC) cluster using 32 cores with a clock speed of 2.4 GHz and 2 GB memory per core.

### Numerical results

Before answering the three research questions in their corresponding sections, we discuss the value of stochastic solution (VSS) and the expected value of perfect information (EVPI). With the considered network and parameter settings, we observe that the first stage solution of the expected value problem yields an infeasible problem when fixed in the original problem. This is caused by the need for storage in some scenarios of the original problem, which is not used in the solution of the expected value problem. As the solution of the expected value problem is infeasible in the original problem, the VSS amounts to $\infty$, which underlines the importance of considering uncertainty in this context. Then, the EVPI amounts to €135k per day, which is a considerable amount of money when aggregated over a year. However, considering that this is only a small fraction of the total revenue, this market with its underlying network and capacity and storage options, seems to cope naturally well with the fact that some demand and prices are uncertain.

#### Risk aversion

##### **Research question 1**

How does risk aversion affect capacity allocation and capacity availability in various strategic and operation stages?

**Fig. 7:**
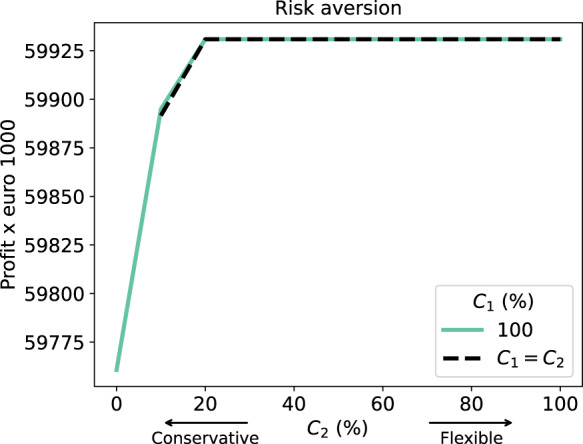
Different levels of risk aversion in the first and second stage

We assumed that the TSO is risk averse and therefore wants to change its decisions as little as possible. To quantify the extent of the TSO’s risk aversion in our model, we have introduced parameters $C_1$ and $C_2$ in Sect. 4. We run the model with different values for $C_1$ and $C_2$ with $C_2 \le C_1$ and show the result in Fig. 7. Based on this result, we make two observations.

First, we see that being slightly less risk averse already yields considerable gains in the system profit. Note that small percentages correspond to considerable amounts of money in this industry, as mentioned in Sect. 1. Additionally, we see that the problem with $C_1=C_2=0$ is infeasible as combining all scenarios into one solution without a recourse action is not possible. We observe that mainly $C_2$ impacts the objective value as the graphs for $C_1 = 100\%$ and $C_1=C_2$ are almost identical.

Second, this figure also has an interpretation relating to realistic first-stage decisions under different market circumstances. If suppliers are unwilling to risk an individual third-stage contract infeasibility, putting them in a vulnerable position when needing a secondary-market deal, the likely optimal first-stage decision will resemble the situation of complete strictness: each supplier will make sure to book an amount of capacity in the first stage that will allow them to fulfill all contractually obligated demand in any scenario. This is system sub-optimal, but no supplier will be at risk of putting themselves in a vulnerable position of having to rely on another supplier to be able to meet their obligations. If there is distrust between suppliers, we end up here: suppliers expect their competitors to extort any vulnerability they might have. However, since we assume perfect competition, suppliers may approve of a first-stage decision that is system optimal in expectation, but requires some suppliers to rely on cooperation of competitors to repair any contractual infeasibilities that may occur in some scenarios by offering the required capacity at a reasonable price.

**Fig. 8:**
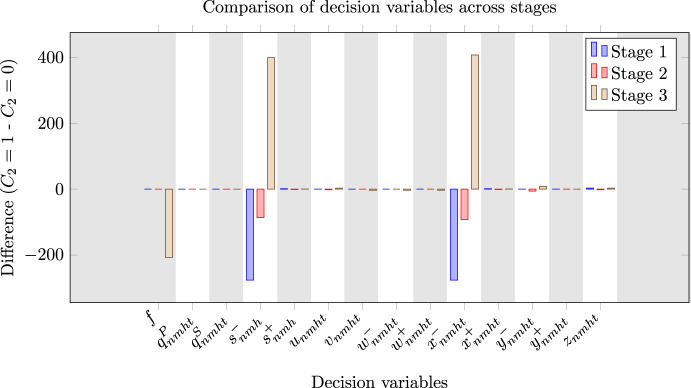
Comparison in decision variables between $C_2 = 100\%$ and $C_2 = 0\%$ while $C_1=100\%$

Additionally, we show the difference in decision variables, aggregated over all indices, between $C_2 = 100\%$ and $C_2 = 0\%$ while $C_1=100\%$ in Fig. 8, which shows that, when the system is less restricted, the system waits with assigning exit capacity to a supplier until the demand and price uncertainties are revealed. Note that the figure only includes decision variables at those stages for which they are also included in the objective function of our method.

#### Bottlenecks

##### **Research question 2**

What are the bottlenecks of the system and how can we solve them?

We consider three bottlenecks: production and pipe capacities and minimum contract volumes. We look at shadow prices of Constraints (2a), (2c), and (2b), respectively, to identify these bottlenecks. Table 5 shows the approximate gains in the objective due to an increase of one MWh on the right hand side of Constraints (2a) and (2c). We sum the shadow prices of the individual capacity constraints corresponding to each scenario and time block to obtain an estimate of how much an capacity increase of one MWh positively impacts the objective function. We observed that three pipes are the most important bottlenecks: Johan Sverdrup Gassror, Vesterled, and Troll Gassror. If we could increase the capacity of these pipes, the system’s overall throughput would increase considerably. More specifically, increasing the capacity of Johan Sverdrup Gassror with one MWh per time block yields approximately €144,000 per day. The rightmost column in Table 5 represents the maximum aggregated shadow prices over the different time blocks. By comparing the second and third column in Table 5, we can get an indication of how the system’s bottlenecks may vary depending on the demands and prices, which vary due to uncertainty and time fluctuations. In some specific scenarios and time blocks, it would be useful to have more capacity in a set of pipes for which the aggregate shadow price is not high. This indicates that the bottlenecks of the system are not static.

**Table 5:** Approximate gains (€) in the objective due to an increase of one MWh on the right hand side of Constraints (2a) and (2c)

Pipe	Total over all time blocks	Maximum
Johan Sverdrup Gassror	144,000	23,040
Vesterled	125,760	20,160
Troll Gassror	124,800	19,200

Node	Total over all time blocks	Maximum
Ekofisk J	144,960	–
Statfjord B	143,040	–
Heidrun	142.080	–
Norne ERB	142.080	–
Skarv ERB	142.080	–
Sleipner A	125,760	–
Ormen Lange A	125,760	–
Aasta Hansteen Plem	124,800	–
Kvitebjorn	124,800	–
Gjøa	123,840	–
Cats Platform	111,360	–

When considering Constraint (2c), we observed that eleven facilities are the most important bottlenecks, which are also listed in Table 5. Increasing the production capacity of these nodes would also improve the system’s throughput considerably. More specifically, as stated in Table 5, increasing the production capacity of Ekofisk J with one MWh per time block yields approximately €144,960 per day. Finally, Figure 14 in Appendix 4 shows the shadow prices of constraint (2b). We observe that the German markets are the most important bottlenecks. Lowering the contracts in these three markets would benefit the system’s profit.

#### Use of storage

##### **Research question 3**

What is the value of booking-flexibility for storage facilities?

To answer this research question, we evaluate the system by applying our model with Constraints (2j) and (2k) relaxed. We then compare this to the solution of our base model and show the results in Table 6. When comparing these two solutions, we see that the (expenses for) storage capacities are about ten times lower when these constraints are relaxed. This shows that there is a considerable added value in having the flexibility to book different storage capacities for different time blocks. Conversely, the system cost of being inflexible in this context is high as well. Furthermore, this finding entails that this flexibility option will be lucrative to suppliers even if the party administering storage would ask a high surcharge. Finally, we see that the relaxed model uses the booked storage capacity more often than the base model, indicating a more efficient system.

**Table 6:** Illustration of the value of booking-flexibility for storage facilities

	Storage capacity used (%)	Total storage capacity acquired (MWh)	Objective (€)
Base model	31.59	109,260	59,931,000
Relaxed model	89.98	1,409,390	60,623,000

### Model scalability

To give an indication of our method’s tractability, we provide more details in Table 7.^1^ For this specific run, we used the settings $C_1=1$ and $C_2=0$. We see that the solver’s presolve method considerably decreases the model size. Additionally, we observe that for this large, but continuous, linear program the solver finds an interior point solution within minutes, which is the case for the configurations used in both Sects. 5.2.2 and 5.2.3. In Sect. 5.2.1, the solution times are much longer as the shadow prices must be computed for which a computationally expensive crossover method is required.

**Table 7:** Properties of a run with our model using $C_1=1$ and $C_2=0$

Attribute		Value
Before presolve	Number of decision variables	13,610,640
	Number of constraints	12,328,888
After presolve	Number of decision variables	4,620,036
	Number of constraints	3,490,493
	Strategic nodes in scenario tree (red nodes in Fig. 1)	121
	Operational nodes in scenario tree (blue nodes in Fig. 1)	968
	Solution time (s)	225

These values give an indication of our method’s tractability

**Fig. 9:**
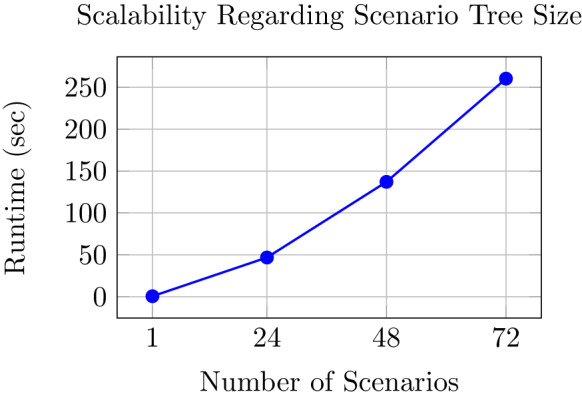
Runtimes (seconds) for different numbers of scenarios. Each datapoint is an average over twelve runs

Figure 9 depicts the runtimes of our model applied to scenario trees of different size, which indicates an exponential but limited growth pattern. The results in this figure were obtained in the following manner: we reduced the original scenario tree by randomly removing branches, solve the smaller problem with our model and report the runtimes. We executed this process twelve times to account for randomness due to random sampling and report the average in Fig. 9.

**Fig. 10:**
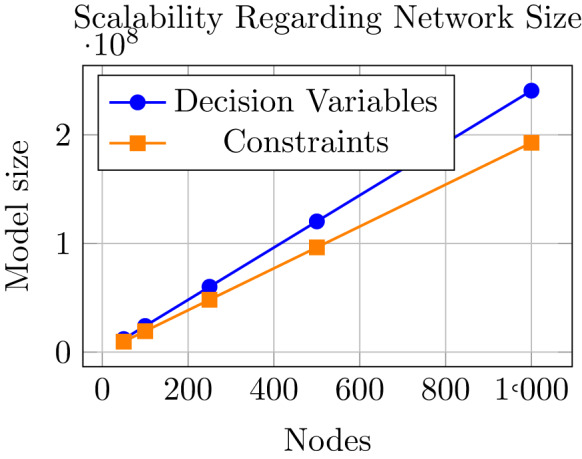
Model size in terms of constraints and decision variables for different (sparse) graphs; $|A| = 2|N|$

**Fig. 12:**
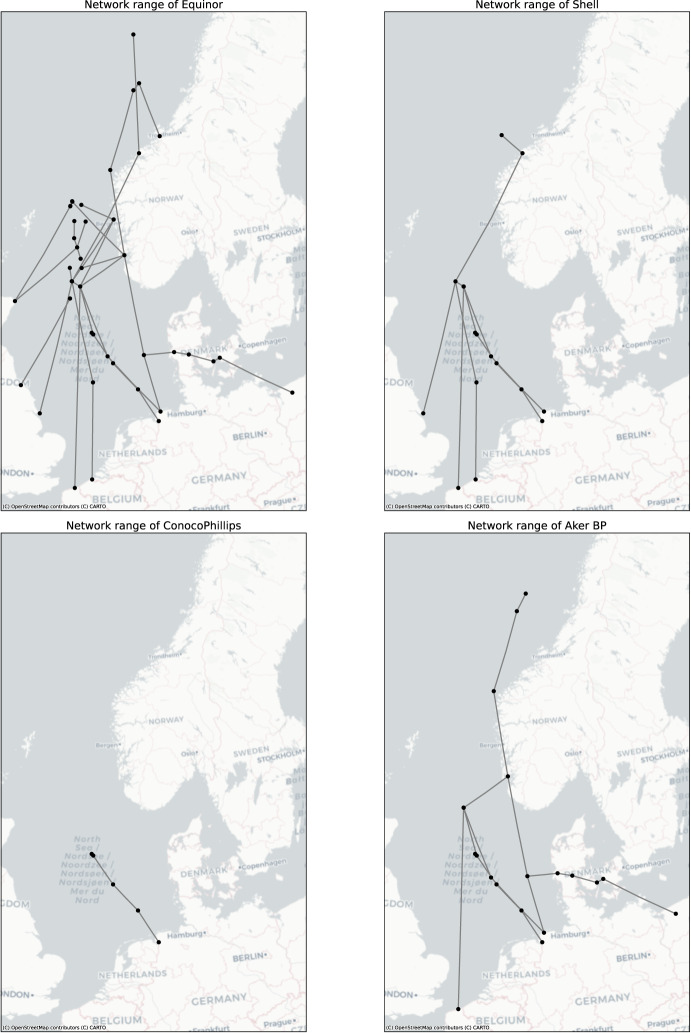
Network ranges of Equinor, Shell, ConocoPhillips, and Aker

**Fig. 13:**
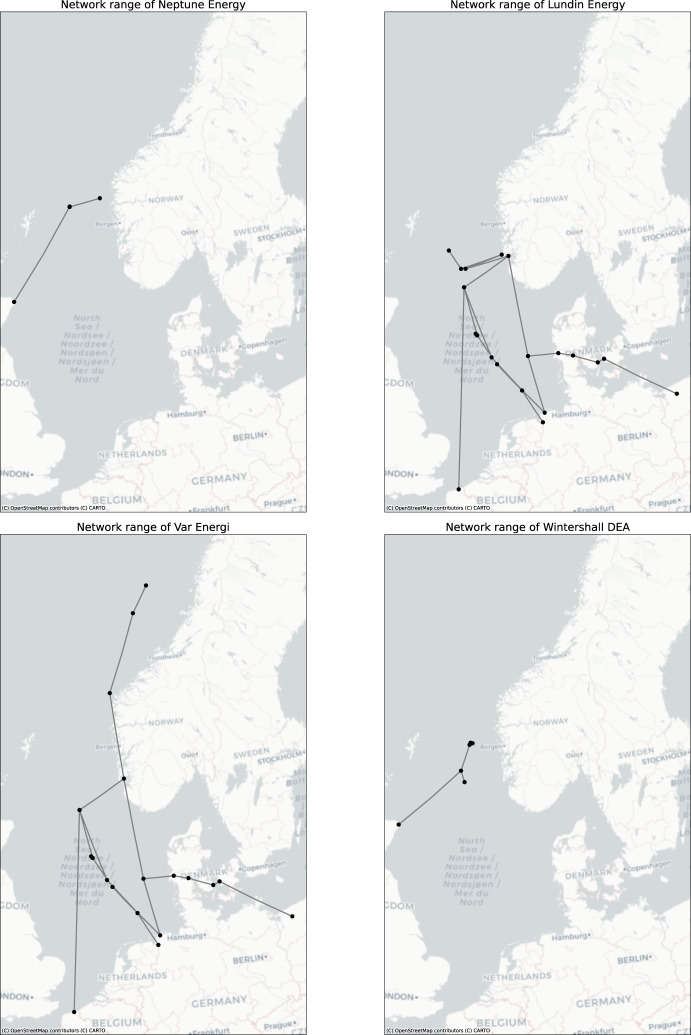
Network ranges of Neptune, Lundin, Var, and Wintershall

**Fig. 14:**
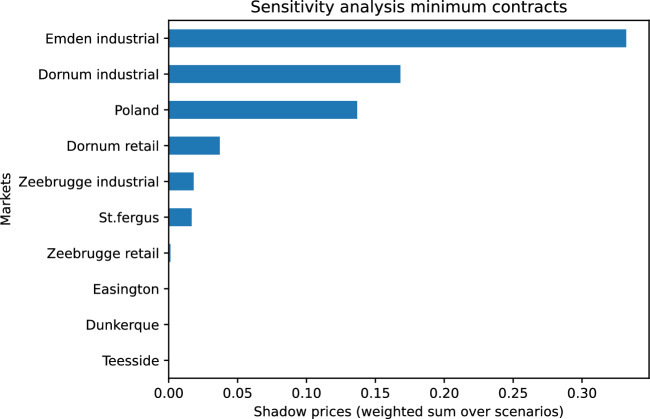
Shadow prices of minimum contracts

As shown in Table 1, the network considered in this study is relatively large compared to other existing literature. However, we would also like to briefly shed the light of the expected performance of our model on networks of even larger scale or networks that are more intricate. To this end, we would like to point out that the size of our model grows linearly with the network size if the other parameters remain the same, as shown in Fig. 10, while the solving time will increase more than linearly but remains manageable. Compared to other models in the literature, our method is still tractable with much larger instances, both in terms of network size and the number of scenarios. However, this will also greatly depend on the network structure and inherent complexities.

### Discussion

While our findings support the hypothesis that the risk aversion negatively affects the entry–exit capacity utilization under uncertainty, certain limitations—mainly caused by made assumptions—should be considered when interpreting these outcomes.

An important asset of our model is its applicability to any gas network with a graph representation of any size. However, the insights on system dynamics presented in this study can only be extended to other gas networks to a limited extent. This is due to the complexity of networks—which we identify as an important driver of these dynamics—highly depending on the maturity and size of a network.

As an example of this, our method could provide considerably more added value when applied to gas networks whose graph representation is not acyclic. Typically, such networks have more possible overlapping routes competing for capacity than the network we considered in this study. As a consequence of this, more profit can be realized from secondary market trading due to optimal flow direction reversal in specific scenarios. A possible application will be to study networks with bidirectional pipelines, such as the Interconnector UK or Bacton Balgzand Line. We conjecture that the gap between a fully risk averse and fully non risk averse TSO will be significantly larger in networks that contain a substantial number of cycles caused by such bidirectional pipelines.

The last few years, decentralized electricity production has increased considerably, which led to a shift in the use of natural gas: as the base load for electricity networks decreases due to the higher volatility of renewable sources, the role of natural gas in stabilizing electricity supply becomes pivotal. Consequently, the demand for natural gas is subject to a new source of stochasticity introduced by supply fluctuations in the electricity market. This underlines the importance of accounting for uncertainty when modeling natural gas markets in the context of securing a stable energy supply in Europe.

Furthermore, novel gaseous commodities, most notably hydrogen, will be used increasingly to make the European energy mix more sustainable. In order to optimally exploit existing infrastructure, these commodities can be injected into the flow of conventional natural gas, directly impacting flow volumes in a network. As hydrogen needs to be cleanly produced, its production facilities should be powered by green electricity sources. This means that, in terms of supply stability, hydrogen production suffers from the same capriciousness mentioned before. Thus, the paradigm of accrediting a central role to uncertainty and flexibility when studying gas networks is also highly valuable in this light.

We observe that the problem formulation from Sect. 3 has similarities with the static stochastic knapsack problem (Steinberg and Parks 1979), which has uncertain values per item. In our case, the knapsack is the combination of all the markets, which has a limit on the sales due to pipe capacities, and the knapsack items are the gas units. The results show that first, the most profitable markets are served, which, among others, depends on their gas prices. In the case of minimum volume contracts, the “knapsack” is already partially filled with mandatory “items”, i.e., gas units.

We assume that suppliers are price takers. Given prices in the markets, the lowest-cost producer has the highest willingness to pay for capacity, which is the implicit mechanism driving social welfare maximization. We have opted for fixed demand and prices, and not inverse demand curves as demand changes caused by the market power dynamics would be hard to decouple from other observed effects. This allows us to focus on how price arbitrage possibilities drive demand for capacity, and capacity flexibility. Including market power in this model could be a topic for future research. Another topic of future research could be including supply and demand dynamics in the gas prices.

The objective of our model produces a first stage that is system optimal but not necessarily agent optimal. This is a consequence of assuming that all agents are price takers. The first stage decision produced by our model does not contain a balancing of interests between the different suppliers, which may bring some suppliers in vulnerable positions because there is no mechanism in place that guarantees a fair distribution of risk over all the suppliers. It is possible that smaller suppliers would have a higher degree of risk aversion. In our current study, suppliers have no individual agency on risks they are willing to take, only system wide risk stances have been addressed.

In our case study, we consider one large and seven small suppliers, which is representative for the actual situation at the Norwegian Continental Shelf where Equinor is by far the largest gas producer. Additionally, the network range of these suppliers differ considerably from each other, as shown in Appendix 3. Therefore, the competition between suppliers on each pipeline is relatively small. This phenomenon influences the results and therefore impacts the generalizability of our findings to other energy markets with different supplier proportions and network structures. Future research could entail testing our model on other gas networks.

As our results are based on data, which mimic reality, our findings are not merely useful for academics, but also for practitioners from the industry. Additionally, our method, a stochastic linear program, could be applied to larger scenario trees or networks.

## Conclusion

This work addresses the optimization of natural gas entry and exit capacity under uncertainty using a multi-stage stochastic programming approach. Given the inherent uncertainties in gas demand and pricing, we introduce a decision-making framework spanning three planning horizons: long term (months ahead), day ahead, and intraday. The optimization framework considers multiple gas suppliers interacting with a Transmission System Operator (TSO). Suppliers book entry and exit capacities and can adjust their positions in a secondary market. The objective is to maximize system-wide profitability while incorporating costs related to capacity booking, production, storage, and gas flow. The physical infrastructure is represented as a directed graph, where gas flow is subject to compression losses.

We find that slightly reducing the TSO’s risk aversion already yields considerable gains in the system profit. We argue that checks and balances in the system are pivotal to this improvement, to decrease additional risk aversion among individual suppliers. Additionally, we identify bottlenecks in the Norwegian gas market with respect to the pipeline and production capacities, and minimum contract volumes. Finally, we study the value of flexibility-booking for storage and show a considerable markup for flexibility in this context. These findings might contribute to securing a stable energy supply in Europe.

For future research, we can include disruptable capacities, and the price dynamics of supply-and-demand in our model. Additionally, we can study a model with more stages if the prices and the demands are more volatile and are only realized shortly in advance. Next, we could study the impact of modeling more realistic risk stances of individual suppliers in the first stage on the tractability and scalability of our model. Finally, the inclusion of hydrogen production, transport, or even infrastructure (Zhang et al. 2023) in our model could also be interesting for future research. Similarly to Huppmann and Egging (2014), we could use study market power exerted across several fuels.

## Figures and Tables

**Fig. 11:**
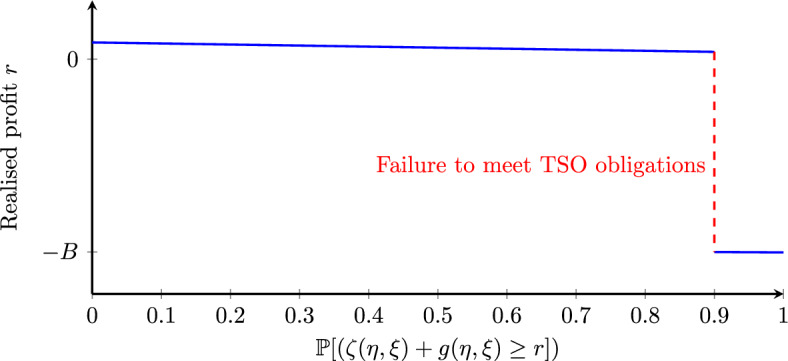
Inverse cdf of the outcome distribution for a given solution and $\eta$

## Data Availability

Data can be found on https://github.com/berendmarkhorst/secondary_gas_market after publication.

## Appendix 1: Methodology

See Tables 8, 9, and 10.

**Table 8:** Sets

Symbol	Description
*K*	Stages; index *k*
*N*	Nodes in the network (production platforms, processing facilities, markets); index *n*
*A*	Arcs/pipes which can transport natural gas; index *a*
$A^{+}_{n}$	Inward arcs into node *n*; index *a*
$A^{-}_{n}$	Outward arcs from node *n*; index *a*
*M*	Scenario tree nodes; index *m*
$M^{(1)}$	Scenario tree nodes corresponding to *long term*; index *m*
$M^{(2)}$	Scenario tree nodes corresponding to *day ahead*; index *m*
$M^{(3)}$	Scenario tree nodes corresponding to *intra day*; index *m*
$\Pi (m)$	All the parent nodes of node *m* and node *m* itself. If $m=1$, then $\Pi (m):= \{1\}$; index $\tilde{m}$
*H*	Time blocks; index *h*
*T*	Suppliers of natural gas; index *t*

**Table 9:** Parameters

Symbol	Description
$c^{P}_{n}$	Production costs in node *n*
$c^{I}_{nmh}$	Storage capacity costs in node *n* in scenario node *m* at time block *h*
$c^{A}_{a}$	Flow costs in arc/pipe *a*
$c^{N+}_{mh}$	Entry capacity costs in scenario node *m* at time block *h*
$c^{N-}_{mh}$	Exit capacity costs in scenario node *m* at time block *h*
$CAP^{P}_{n}$	Production capacity in node *n*
$CAP^{I}_{n}$	Storage capacity in node *n*
$CAP^{A}_{a}$	Flow capacity at arc/pipe *a*
$l_{a}$	Loss rate for flow over arcs/pipes
$p_{m}$	Weight for scenario node *m*, based on the probability and the time block this node represents
$\xi _{nmht}$	Uncertain, minimum demand volume of natural gas in node *n* and scenario node *m* at time block *h* for supplier *t*
$r_{nmh}$	The price of natural gas at scenario node *m* at time block *h* in node *n*
$C_1$	Maximum difference (%) between the first and third stage decisions
$C_2$	Maximum difference (%) between the second and third stage decisions

**Table 10:** Decision variables

Symbol	Description
$x^{+}_{nmht}$	Entry capacity bought at node *n* by the supplier *t* in scenario node *m* at time block *h*
$x^{-}_{nmht}$	Exit capacity bought at node *n* by the supplier *t* in scenario node *m* at time block *h*
$y^{+}_{nmht}$	Entry capacity sold at node *n* by the supplier *t* in scenario node *m* at time block *h*
$y^{-}_{nmht}$	Exit capacity sold at node *n* by the supplier *t* in scenario node *m* at time block *h*
$s^{+}_{nmh}$	Entry capacity sold by the TSO in scenario node *m* at time block *h* at node *n*
$s^{-}_{nmh}$	Exit capacity sold by the TSO in scenario node *m* at time block *h* at node *n*
$f_{mhat}$	Supplier infrastructure flow for supplier *t* at arc *a* in scenario node *m* at time block *h*
$q^{S}_{nmht}$	The volume of gas sold by supplier *t* at node *n* in scenario node *m* at time block *h*
$q^{P}_{nmht}$	The volume of gas produced by supplier *t* at node *n* in scenario node *m* at time block *h*
$v_{nmht}$	The volume of natural gas stored by supplier *t* at node *n* in scenario node *m* at time block *h*
$w^{+}_{nmht}$	The volume of natural gas put into storage by supplier *t* at node *n* in scenario node *m* at time block *h*
$w^{-}_{nmht}$	The volume of natural gas retrieved out of storage by supplier *t* at node *n* in scenario node *m* at time block *h*
$z_{nmht}$	Storage capacity bought at node *n* by the supplier *t* in scenario node *m* at time block *h*
$u_{nmht}$	Storage capacity sold at node *n* by the supplier *t* in scenario node *m* at time block *h*
$\lambda ^s_{nmht}$	Volume of entry ($s=+$) or exit ($s=-$) capacity at node *n* supplier *t* has acquired so far up to scenario node *m* in time block *h*
$\kappa _{nmht}$	Volume of storage capacity at node *n* supplier *t* has acquired so far up to scenario node *m* in time block *h*
$\gamma _{{k}mhat}$	The difference in flow decisions between the third and the corresponding first (${k}=1$) or second stage (${k}=2$) decision of trader *t* at arc *a* in scenario node *m* and time block *h*
$\delta ^s_{{k}nmht}$	The difference in acquired entry ($s=+$) or exit ($s=-$) capacity between the third and the corresponding first (${k}=1$) or second stage (${k}=2$) decision of trader *t* at node *n* in scenario node *m* and time block *h*
$\theta _{{k}nmht}$	The difference in acquired storage capacity between the third and the corresponding first (${k}=1$) or second stage (${k}=2$) decision of trader *t* at node *n* in scenario node *m* and time block *h*

## Appendix 2: Relation between risk aversion constraints and (C)VaR

In order to understand how the relation between risk aversion constraints and (C)VaR manifests itself in our problem context, let us assume that, in case of failure to facilitate the guaranteed transportation plan, the TSO incurs a penalty *B*, which is much larger than the best attainable social welfare. We let $g(\eta , \xi )$ denote the possible penalty incurred for a specific $\eta$ and $\xi$, such that $g(\eta , \xi )$ is either 0 or $-B$. Additionally, we let $\zeta (\eta , \xi )$ be the realized system profit, akin to Eq. (1), for a given realization of the uncertainty $\xi$ and decision $\eta$.

One has to take into account that $\eta$ may constrain the rest of the variables in the program, and therefore the optimal objective. Yet, due to the gravity of the consequences of a failure, it is reasonable to assume that the incurred penalty *B* will dominate differences in the objective caused by $\eta$ constraining other variables:

$$\max _{\eta , \tilde{\eta }} | \zeta (\eta , \xi ) - \zeta (\tilde{\eta }, \xi )| \ll B \; \forall \xi .$$

An example of this setting is illustrated by Fig. 11, which shows the inverse cumulative distribution function (cdf) of the realized profit for a given solution and $\eta$, with the large jump separating the situations where failure does and does not occur.

As there exists an equivalence relation between chance constraints and VaR constraints (Sarykalin et al. 2008), we can say that adding such a chance constraint is equivalent to constraining the VaR of $\zeta (\eta , \xi )+g(\eta ,\xi )$ (for example, to be above $\frac{-B}{2}$). Because the $\alpha$-chance constraint directly controls the fraction of scenarios in which a penalty occurs, i.e., the location of the jump in Fig. 11, it ensures that $\text {VaR}_\alpha \left( \zeta (\eta , \xi ) + g(\eta , \xi ) \right) \gg -B$. Furthermore, for all $\beta < \alpha$ the following holds:

$$\begin{aligned} \text {CVaR}_\beta \ge \frac{\alpha }{1-\beta } \cdot - B \end{aligned}$$

because we may assume in our context that, so lang as $\alpha> \epsilon$ with $\epsilon$ being a very small number, a non-negative feasible solution to our original problem exists for some $\eta$ satisfying the $\alpha$-chance constraint.

## Appendix 3: Network ranges suppliers

See Figs. 12 and 13.

## Appendix 4: Bottlenecks

See Fig. 14.

## Footnotes

1 Note that the reported number of constraints and decision variables slightly differs from the theoretical numbers due to implementation choices.
